# Effect of Coptidis Rhizoma on gastrointestinal system before and after processing with wine based on gut microbiota and short chain fatty acids

**DOI:** 10.3389/fphar.2024.1492047

**Published:** 2024-11-18

**Authors:** Rui Tian, Wen-Xiao Wang, Ya-Ya Bai, Yu-Ping Tang, Qiao Zhang, Shi-Jun Yue

**Affiliations:** ^1^ Key Laboratory of Shaanxi Administration of Traditional Chinese Medicine for TCM Compatibility, State Key Laboratory of Research and Development of Characteristic Qin Medicine Resources (Cultivation), Shaanxi Collaborative Innovation Center of Chinese Medicinal Resources Industrialization, Shaanxi Traditional Chinese Medicine Processing Technology Heritage Base, Shaanxi University of Chinese Medicine, Xi’an, China; ^2^ International Joint Research Center on Resource Utilization and Quality Evaluation of Traditional Chinese Medicine of Hebei Province, Hebei University of Chinese Medicine, Shijiazhuang, China

**Keywords:** Coptis deltoidea, wine-processing, gut microbiota, short chain fatty acids, gastrointestinal system

## Abstract

**Background:**

Coptis deltoidea C.Y. Cheng et Hsiao (CD), commonly used in the treatment of heat-toxin congestion and excessiveness. However, CD needs to be processed with wine for alleviating the bitter and cold of CD, meanwhile, reducing the gastrointestinal damage. The research assessed the discrepant effects of CD on gastrointestinal system before and after processing with wine, and explore the potential mechanisms.

**Methods:**

The ingredients in CD and CD processed with wine (PCD) were performed on Ultra Performance Liquid Chromatography Mass Spectrometry (UPLC-MS). The mice were treated with CD and PCD once a day for 6 weeks (0.65 and 2.6 g/kg, i.g.). The pathological changes of gastrointestinal tract were evaluated, and the serum inflammatory factors and Nuclear Factor kappa-light-chain-enhancer of activated B cells (NF-κB) RelA (p65) protein of tissues were determined. The short chain fatty acids (SCFAs) of feces were analyzed by UPLC-MS, the gut microbiota (GM) changes were performed on 16 S rRNA sequencing.

**Results:**

Ingredients analysis declared that the alkaloids, flavonoids, phenylpropanoid compounds were the main metabolites in CD and PCD. CD reduced body weight and food intake, and the effect of CD on fecal water content increased first and then decreased with the prolongation of administration time, while its effect on intestinal transport time was exactly the opposite, reduced the SCFAs contents of feces. CD caused different degree of damage to the gastrointestinal tract, and the effect on the small intestine and colon was more obvious, which increased the expression of NF-κB p65 and elevated the inflammatory factors levels. PCD were weaker than that of CD. In addition, CD and PCD can change the composition of GM, and reduced the levels of *Lactobacillus*, *Allobaculum*, *Ruminococcus*, and *norank_f_S24-7*, increased the levels of *Akkermansia*, *Dorea*, *Bacteroides*, and *Blautia* at the genus level. However, PCD induced a milder effect of GM dysregulation than that of CD.

**Conclusion:**

CD can cause damage to the gastrointestinal tract, which may be related to the GM disorders, SCFAs changes-mediated by GM, abnormal NF-κB p65 expression and increased inflammatory factors levels, interestingly, PCD had a lower effect than CD, which may be related to the differences in the types and contents of ingredients in CD after processing. And this study provided data support for the mechanism of processing with wine to alleviate “bitter-cold injury the stomach” of CD.

## 1 Introduction

Traditional Chinese medicines (TCM) have been widely used for the clinical treatment of various diseases in countries such as China, South Korea and Japan, and their effectiveness has been confirmed for thousands of years. Coptidis Rhizoma is the dried rhizome and root of *Coptis chinensis* Franch., *Coptis deltoidea* C.Y. Cheng et Hsiao., and *Coptis teeta* Wall., is known for its ability to clear heat, remove fire and detoxify. It can be used to treat dampness and heat, vomiting, diarrhea, jaundice, high fever, dizziness and toothache (China Pharmacopeia Commission., 2020). It has demonstrated a wide range of pharmacological effects that primarily include hypoglycemic, antibacterial, antioxidant, anti-inflammatory, and antitumor properties (Song et al., 2020; Wang et al., 2019). Coptidis Rhizoma has the characteristic of bitter taste and cold nature, and traditional Chinese medicine theory suggests that the bitter and cold of TCM can injure the stomach.

It is important to note that the term “stomach,” as referenced in TCM theory and the clinical manifestations associated with “injury to the stomach,” extends beyond its anatomical definition. It encompasses not only the stomach itself but also includes both the large and small intestines, along with other components of the digestive system. This concept is supported by a passage from Ling Shu-Ben Shu, which states: “The large and small intestines all belong to the stomach” (Bai et al., 2023). In addition, experimental studies have shown that large doses of bitter cold Chinese medicines can caused gastrointestinal tissue damage in animals, some of which can lead to death (Li., 2005). Surprisingly, previous literature has shown that processing with wine can reduce the bitter and cold nature of Coptidis Rhizoma and relieve the damage to intestinal tissue (Zhou et al., 2023).

TCM were typically processed to enhance its efficacy, mitigate or eliminate toxicity and side effects, and ensure clinical applicability (Pei et al., 2022; Zhong et al., 2019). The methods of processing TCM with wine had been documented since the Han Dynasty, and Coptidis Rhizoma processed with wine was first recorded in Qian Jin Yi Fang. Until the 2020 edition of the Chinese Pharmacopoeia, stir-frying with wine was recognized as one of the primary methods for moderating the bitter-cold properties of Coptidis Rhizoma. Existing studies have confirmed that high doses of Coptidis Rhizoma can adversely affect the gastrointestinal tract, leading to issues such as constipation. However, the mechanisms by which processing Coptidis Rhizoma with rice wine mitigates its coldness and its specific effects on the gastrointestinal system remain unclear. Therefore, further studies are needed.

Gut microbiota (GM) was considered to be a “forgotten organ” and helpful for the body’s nutrient absorption, drug metabolism, immune regulation, maintains the structural integrity of the intestinal mucosal barrier and inhibits pathogens (Flint et al., 2012; Jandhyala et al., 2015; Zhang Q. et al., 2023). Under normal physiological conditions, the types and proportions of GM were in homeostatic equilibrium, and play the functions of immunity, metabolism, and maintenance of intestinal barrier homeostasis (Yue et al., 2019). Under pathological conditions, the micro-ecosystems within the intestinal tract are significantly compromised, resulting in an increase in the population of conditionally pathogenic bacteria and a decrease in probiotic bacteria. This imbalance leads to GM dysbiosis, which is closely associated with the development of various gastrointestinal diseases, including diarrhea, inflammatory bowel disease, metabolic disorders (such as obesity and diabetes mellitus), and cardiovascular diseases (Afzaal et al., 2022; Nishida et al., 2018).

Short-chain fatty acids (SCFAs) are the primary metabolites of GM. They played a crucial role in promoting the growth of beneficial bacteria, improving GM composition, and regulating host immune balance (Liu et al., 2021; Tan et al., 2014). Current research has demonstrated that acetic, propionic, and butyric acids constitute over 95% of SCFAs present in the human gut. Furthermore, Firmicutes is identified as the predominant species within the gut microbiota responsible for SCFA production (Louis et al., 2014). Moreover, the occurrence of gastrointestinal diseases was closely related to GM changes (Dong et al., 2019; Li et al., 2020). For instance, the relative abundance of *Bifidobacteria* and *Lactobacillus* were reduced and *Bacteroides* were increased in adults with constipation compared to controls (Chassard et al., 2012; Khalif et al., 2005). Meanwhile, constipated patients had lower SCFAs than healthy controls. Furthermore, the intake of probiotics can alleviate constipation by regulating the composition of the GM and the production of SCFAs (Jeong et al., 2023; Lai et al., 2023). Fascinatingly, the regulatory effect of probiotics on intestinal motility were also related to neurotransmitters (Dimidi et al., 2017). Thus, more attention to GM may help to reveal the pathogenesis of gastrointestinal diseases.

In this study, the components change of Coptidis Rhizoma before and after processing with wine were studied, as well as effects on GM and SCFAs were studied, simultaneously, the pathological effects of the gastrointestinal tract were observed, the levels of inflammatory factors and the expression of NF-κB p65 were analyzed, aiming to explore the potential mechanism of Coptidis Rhizoma processing with wine in alleviating side effects on the intestines, and provide scientific evidence for its rational clinical application.

## 2 Materials and methods

### 2.1 Chemicals and reagents

4% paraformaldehyde solution was purchased from Shaanxi Zhonghui Hecai Biomedical Technology Co., Ltd. (Shaanxi, China). 75% ethanol was bought from Tianjin Kemio Chemical Reagent Co., Ltd. (Tianjin, China). Carmine dye was obtained from Sigma Aldrich.

### 2.2 Plant material

*Coptis deltoidea* C.Y. Cheng et Hsiao (CD) were purchased from Shaanxi Shangluo Panlong Botanical Pharmaceutical Co., Ltd. (Shaanxi, China), and identified by Yonggang Yan (the professor of Shaanxi University of Chinese Medicine). The voucher specimens (No. 20220301) were deposited in Shaanxi University of Chinese Medicine. The preparation of CD processed with wine (PCD) was as follows: PCD was made up of CD slices mixed with yellow rice wine (100:12.5), slightly moistened, waiting for yellow rice wine to be absorbed, and then placed in a container (150 ± 10) °C until slightly scorched spots appeared on the surface of CD, then, PCD was obtained (China Pharmacopeia Commission., 2020). Take the appropriate amount of CD and PCD add 10 times the amount of water, soak for 30 min at room temperature and then decoct, keep slightly boiling for 30 min, filtered while hot; the remaining dregs of the drug add 8 times the amount of water and decoct, keep slightly boiling for 30 min, filtered while hot, and then combine the two decoctions of the resulting filtrate, concentrated, cooled to room temperature, and then placed in a freeze dryer machine freeze-dried to a powder, stored in −20°C refrigerator for backup.

### 2.3 UPLC-ESI-MS/MS analysis

CD or PCD powder (50 mg) was accurately weighed and add 1,200 μL of - 20°C pre-cooled 70% methanolic aqueous internal standard extract (less than 50 mg added at the rate of 1,200 μL extractant per 50 mg sample). Vortex once every 30 min for 30 s, for a total of 6 times. After centrifugation (rotation speed 12,000 rpm, 3 min), the supernatant was aspirated, and the sample was filtered through a microporous membrane (0.22 μm pore size). The sample extracts were analyzed using an UPLC-ESI-MS/MS system and Tandem mass spectrometry system. The analytical conditions were as follows, UPLC: column, Agilent SB-C18 (1.8 µm, 2.1 mm * 100 mm); The mobile phase was consisted of solvent A, pure water with 0.1% formic acid, and solvent B, acetonitrile with 0.1% formic acid. Sample measurements were performed with a gradient program that employed the starting conditions of 95% A, 5% B. Within 9 min, a linear gradient to 5% A, 95% B was programmed, and a composition of 5% A, 95% B was kept for 1 min. Subsequently, a composition of 95% A, 5.0% B was adjusted within 1.1 min and kept for 2.9 min. The flow velocity was set as 0.35 mL per minute; The column oven was set to 40°C; The injection volume was 2 μL. The effluent was alternatively connected to an ESI-triple quadrupole-linear ion trap (QTRAP)-MS. The ESI source operation parameters were as follows: source temperature 500°C; ion spray voltage (IS) 5,500 V (positive ion mode)/- 4,500 V (negative ion mode); ion source gas I (GSI), gas II (GSII) and curtain gas (CUR) were set at 50, 60, and 25 psi, respectively; the collision-activated dissociation (CAD) was high. QQQ scans were acquired as multiple reaction mode (MRM) experiments with collision gas (nitrogen) set to medium. DP (declustering potential) and CE (collision energy) for individual MRM transitions was done with further DP and CE optimization. A specific set of MRM transitions were monitored for each period according to the metabolites eluted within this period.

### 2.4 Animal experiments

Sixty male C57BL/6 mice (18–22 g) were purchased from Xi’an Keaoke Biotechnology Co., Ltd. (Xi’an, China. License No: SCXK 2021-0006). All animal experiments were approved by the Animal Experimental Ethical Committee of Shaanxi University of Chinese Medicine (Approval number: SUCMDL20210310005). The mice were housed at 21°C, 55%–60% ambient humidity under 12 h light (08:00 to 20:00):12 h dark conditions. Mice were randomly divided normal control (NC), CD, high-dose CD (CD-H), PCD and high-dose PCD (PCD-H) groups after adaptively feeding for 1 week, with 12 mice in each group. Mice in CD and CD-H groups were treated with CD (i.g., 0.65 and 2.6 g/kg), respectively, and mice in PCD and PCD-H groups were treated with PCD (i.g., 0.65 and 2.6 g/kg), respectively, and NC group was treated with an equal volume of physiological saline, 6 consecutive weeks, once a day.

### 2.5 Determination of body weight, food intake, fecal water content and intestinal transit time in each group of mice

The body weight, food intake, fecal water content, and intestinal transit time of mice were measured in the first, third and sixth weeks. The intestinal transit time was measured in the following ways: Each mouse was transferred to a clean empty cage and adapted for 1 h, and treated with 150 μL of Sigma-Aldrich solution (i.g.), then, the production of red fecal particles was monitored every 10 min starting 120 min after administration. The collected stools were weighed and dried at 95°C, and the fecal water content was calculated as the following formula:

$$\text{Fecal water content }\left(\%\right)=\left(\text{wet weight}-\text{dry weight}\right)/\text{wet weight}\times 100\%.$$

### 2.6 Histopathological examination

As the previous study description (Zhang Q. et al., 2021), stomach, small intestine and colon tissues were fixed in 4% paraformaldehyde solution, and stained with hematoxylin-eosin (HE). The histopathological changes were observed by a light microscope (Nikon eclipsets 2, Japan).

### 2.7 Detection of interleukin-6 (IL-6), interferon gamma (IFN-γ) and tumor necrosis factor alpha (TNF-α)

The concentrations of total protein were determined using a bicinchoninic acid (BCA, No. BC0524) protein assay kit (Shaanxi Zhonghui, Xi’an, China), and the levels of IL-6, IFN-γ, and TNF-α (No. 20220620, 20220618, 20220724) in serum were measured using enzyme-linked immunosorbent assay (ELISA) kits (Meimian, Yancheng, China).

### 2.8 IHC analysis

As described in the previous literature (Zhang Q. et al., 2021), Paraffin-embedded small intestine sections were processed and added with primary antibody (NF-κB p65 1:200 dilution, No: AF5006, Affinity, China; TLR4 1:100 dilution, No: GB11519, SERVICEBIO, China) overnight at 4°C, and washed three times with PBS and incubated with Goat anti-Rabbit IgG (Dilution: 1:500, No: BL732A, BIOSS, China) for 40 min at 37°C, the slices were stained with DAB and subsequently washed to visualize the target signals. The measurement of mean density was performed on Image-Pro Plus 6.0 software (Media Cybernetics, Silver Spring, MD, United States).

### 2.9 WB analysis

As described in the previous literature (Zhang Q. et al., 2021), the sample was processed and 50 μg protein sample was separated by 10% SDS-PAGE gel and the electrophoresis was transferred to the PVDF membrane. TBST was sealed with 5% protein sealing dry powder for 1.5 h, the closure was completed, and TBST was washed three times. Antibodies against β-actin (Dilution: 1:2000; No: BST17353873, BOSTER, China), NF-κB p65 (Dilution: 1:1,000; No: 8242S; CST, United States) and NF-κB p-p65 (Dilution: 1:1,000; No: 3033S; CST, United States) were added and incubated overnight at 4°C. After completion, Goat anti-Rabbit IgG (Dilution: 1:5000, No: BOSTER, BST18J20B18K54, China) was added and incubated for 1 h. The antigen-antibody complex was detected by ECL reagent. Then the gray values of each protein band were determined and analyzed by ImageJ image analysis software.

### 2.10 Analysis of SCFAs in feces

The feces were collected after final administration, and stored at −80°C for further research. An ultra-high performance liquid chromatography coupled with mass spectrometry (UHPLC-MS/MS) system (Vanquish™ Flex UHPLC-TSQ Altis™, Thermo Scientific Corp., Germany) was used to quantitate SCFA in Novogene Co., Ltd. (Beijing, China). Separation was performed on a Waters ACQUITY UPLC BEH C18 column (2.1 × 100 mm, 1.7 μm) which was maintained at 40°C. The mobile phase, consisting of 10 mM ammonium acetate in water (solvent A) and acetonitrile: isopropanol (1:1) (solvent B), was delivered at a flow rate of 0.30 mL/min. The mass spectrometer was operated in negative MRM. Parameters were as follows: IonSpray Voltage (−4500 V), Sheath Gas (35 psi), Ion Source Temp (550°C), Auxiliary Gas (50psi), Collision Gas (55psi).

### 2.11 16 S rRNA gene sequencing

DNA from the fecal samples was extracted, and sequenced by Majorbio biotechnology platform. The extracted DNA was detected by 1% agarose gel electrophoresis. PCR products were detected and quantified using the QuantiFluor-ST™ Blue Fluor Quantitative System. The gene sequencing was performed by Shanghai Meiji Biomedical Technology Co., Ltd. (Contract No: MJ20220916022) and analyzed by Majorbio biotechnology platform.

### 2.12 Statistical analysis

Statistical analysis was performed by GraphPad Prism 8.3.0 and the data were presented as mean ± standard deviations (SD). Differences were considered statistically significant for *p* < 0.05, *p* < 0.01, and *P* < 0.001.

## 3 Results

### 3.1 UPLC-ESI-MS/MS analysis of CD and PCD

The metabolites of CD and PCD were analyzed by UPLC-ESI-MS/MS (Supplementary Figure S1). As showed in Table 1, the results declared that total of 22 metabolites were identified, and the alkaloids, flavonoids, phenylpropanoid compounds were the main metabolites in CD and PCD. Among them, metabolite 17 were not detected in CD, the relative peak area of metabolites 2, 6, 7, 8, 16, 18 and 20 were decreased in PCD, and the others were increased.

**TABLE 1:** Mass spectral data of the characterized metabolites of CD and PCD by UPLC-ESI-MS/MS.

No.	Compound name	Remark	Molecular formula	M/Z	MS^2^	Source		Relative peak area	Reference
						CD	PCD		
1	Epiberberine	[M]+	C_20_H_18_NO_4_^+^	336.123	320, 292	+	+	up	Cheng et al. (2023)
2	(S)-Canadine	[M + H]+	C_20_H_21_NO_4_	339.1471	310, 325, 340, 224	+	+	down	Wang X. L et al. (2023)
3	Dehydrocorydaline	[M]+	C_22_H_24_NO_4_+	366.1701	350, 336, 322, 308	+	+	up	Ma et al. (2024)
4	Dihydrosanguinarine	[M + H]^+^	C_20_H_15_NO_4_	334.1071	306, 304, 290	+	+	up	Ma et al. (2024)
5	Columbamine	[M]+	C_20_H_20_NO_4_^+^	338.1387	338, 323, 322, 308, 294, 279	+	+	up	Ma et al. (2024)
6	Wogonin	[M + H]^+^	C_16_H_12_O_5_	284.0685	285, 211, 179, 167	+	+	down	Hao Y et al. (2020)
7	Ferulic acid	[M + H]^+^	C_10_H_10_O_4_	194.0579	177, 149, 131, 121, 117, 103	+	+	down	Hao Y. M et al. (2020)
8	Vanillic acid	[M-H]^−^	C_8_H_8_O_4_	168.0423	167, 152, 108	+	+	down	Hao Y et al. (2020)
9	Berberine	[M + H]^+^	C_20_H_18_NO_4_	336.1209	321, 320, 318, 306, 304, 292, 278, 275	+	+	up	Hao Y. M et al. (2020)
10	Palmatine	[M-H]^+^	C_21_H_22_NO_4_^+^	352.1544	337, 336, 322, 320, 308, 294, 292, 291	+	+	up	Hao Y et al. (2020)
11	Coptisine	[M]^+^	C_19_H_14_NO_4_	320.0924	292, 277, 262, 249	+	+	up	Hao Y. M et al. (2020)
12	Beberrubine	[M]^+^	C_19_H_16_NO_4_	322.1071	307, 279, 250	+	+	up	Hao Y et al. (2020)
13	Jatrorrhizine	[M]^+^	C_20_H_20_NO_4_	338.1381	322, 308, 294, 280	+	+	up	Hao Y.M et al. (2020)
14	Acacetin	[M + H]^+^	C_16_H_12_O_5_	284.0685	242, 153	+	+	up	Zhang Y et al. (2021)
15	Luteolin	[M-H]^−^	C_15_H_10_O_6_	286.0477	285, 267, 200	+	+	up	Hao Y et al. (2020)
16	Salidroside	[M-H]^−^	C_14_H_20_O_7_	300.1209	299, 179, 119	+	+	down	Hao Y. M et al. (2020)
17	Cryptochlorogenic acid	[M-H]^−^	C_16_H_18_O_9_	354.0951	353, 309, 191	-	+	up	Wang et al. (2022)
18	Caffeic acid	[M-H]^−^	C_9_H_8_O_4_	180.0423	181, 163, 135, 117	+	+	down	Wang et al. (2022)
19	Chlorogenic acid	[M-H]^−^	C_16_H_18_O_9_	354.0951	355, 163, 145	+	+	up	Wang et al. (2022)
20	Protocatechualdehyde	[M-H]^−^	C_7_H_6_O_3_	138.0317	136, 135, 108	+	+	down	Wang X. L. et al. (2023)
21	Magnoflorine	[M]^+^	C_20_H_24_NO_4_^+^	342.1718	299, 282, 265, 237, 222, 207, 191	+	+	up	Wang K. et al. (2023)
22	8-Oxyberberine	[M + H]^+^	C_20_H_17_NO_5_	351.1107	337, 336, 319, 308, 292, 291, 278	+	+	up	Wang et al. (2020)

### 3.2 Effects of CD and PCD on physiological parameters in mice

**FIGURE 1:**
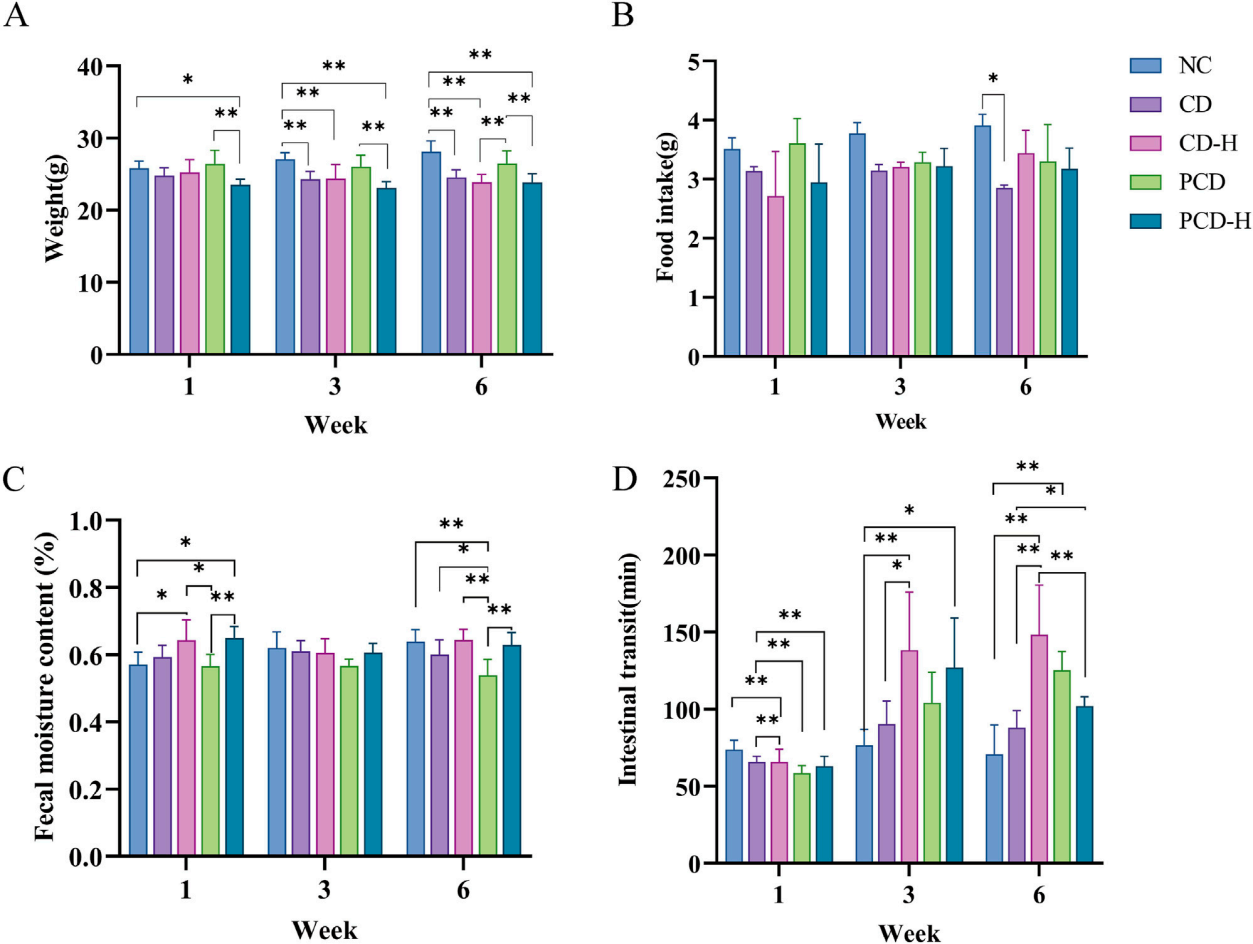
Effects of CD and PCD on physiological parameters in mice. **(A)** weight. **(B)** food intake. **(C)** fecal moisture content. **(D)** intestinal transit of mice. Data were represented as the mean ± SD (n = 6). *p < 0.05, **p < 0.01.

As shown in Figure 1A, compared to NC group, CD and PCD could significantly reduce the weight gain, and the effect of CD was slightly stronger than that of PCD. Similarly, the food intake of mice in CD group at week 6 was significantly lower than that of NC group (*P* < 0.05). And the effect of PCD group was not significant (Figure 1B). Food intake between the first and third weeks among different group shown no obvious change. As shown in Figure 1C, in the first week, administration of CD and PCD could increase the fecal water content compared with the NC group, especially in the high dose group. However, with the prolongation of the administration time, the fecal water content showed a decreasing trend. This indicated that diarrhea occurs after the administration of the drug, and then with the prolongation of the administration time, the fecal water content gradually decreases and then constipation occurs, in which the PCD can alleviate the effect of CD. In Figure 1D, administration of CD and PCD could decrease the intestinal transit time at the first week compared with the NC group. However, the intestinal transit time of healthy mice increased to varying degrees with prolongation of medication.

### 3.3 Effect of CD and PCD on pathological changes in the mouse stomach

**FIGURE 2:**
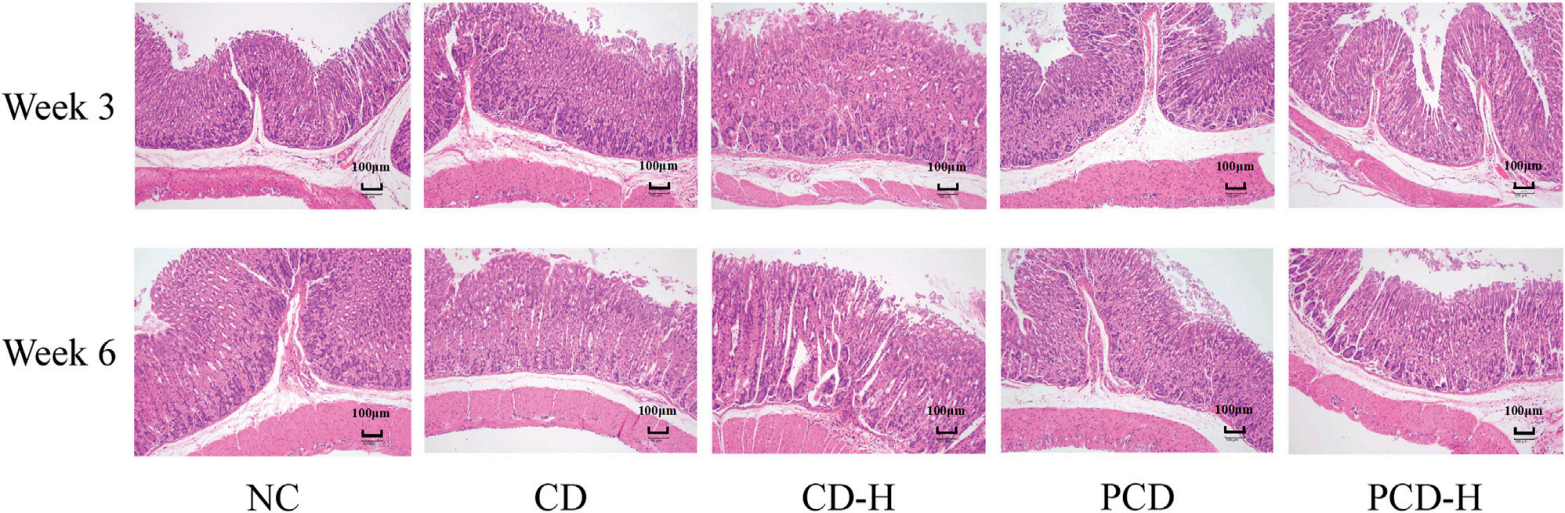
Effects of CD and PCD on pathological changes in the stomach of mice. (Magnification: ×100) (n = 6).

In Figure 2, the mucosal, submucosal, muscularis and serous layer of the gastric tissue of the NC group were structurally intact. After 3 weeks of administration there was a small amount of epithelial cell detachment from the gastric tissue in the CD and PCD groups compared to the NC group. After 6 weeks of administration, epithelial cells were shed in the administered group, and a small infiltration of inflammatory cells was seen in the submucosa of the gastric mucosa in the CD-H group.

### 3.4 Effects of CD and PCD on small intestine of pathological changes

**FIGURE 3:**
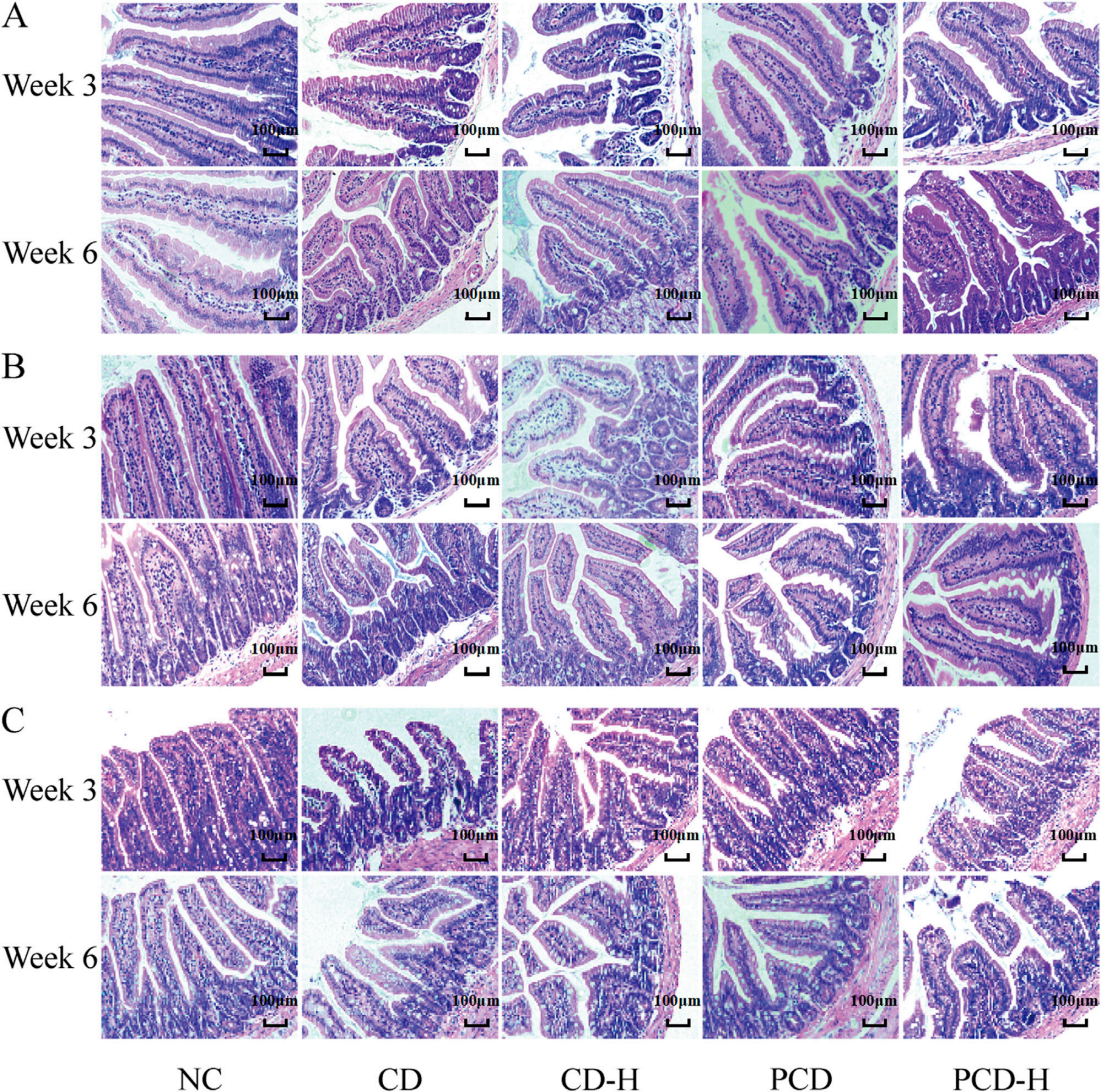
Effects of CD and PCD on pathological changes in the small intestine in mice. **(A)** Duodenum. **(B)** Jejunum. **(C)** Ileum. (Magnification: ×100) (n = 6).

It can be seen from the HE stained sections in Figure 3, that the duodenal villi of the NC group were showed irregular leaf-like or columnar shapes, the ileal villi were showed tongue-like or leaf-shaped, and the villi of the jejunum were shorter and narrower, resembling finger-like projections. After 3 weeks of administration, it was observed that the villi in the small intestine were disrupted, with disorganized arrangement of the mucosal cells. Edema was present, along with partial cell necrosis and shedding. In addition, inflammatory molecules infiltrated the tissue. After 6 weeks of administration, the epithelial cells of the small intestinal villi showed swelling, necrosis, and shedding. The arrangement of the villi became disordered and their length and width were shortened. Additionally, a small amount of inflammatory cell infiltration was observed.

### 3.5 Effect of CD and PCD on the length of colon and cecum in mice

**FIGURE 4:**
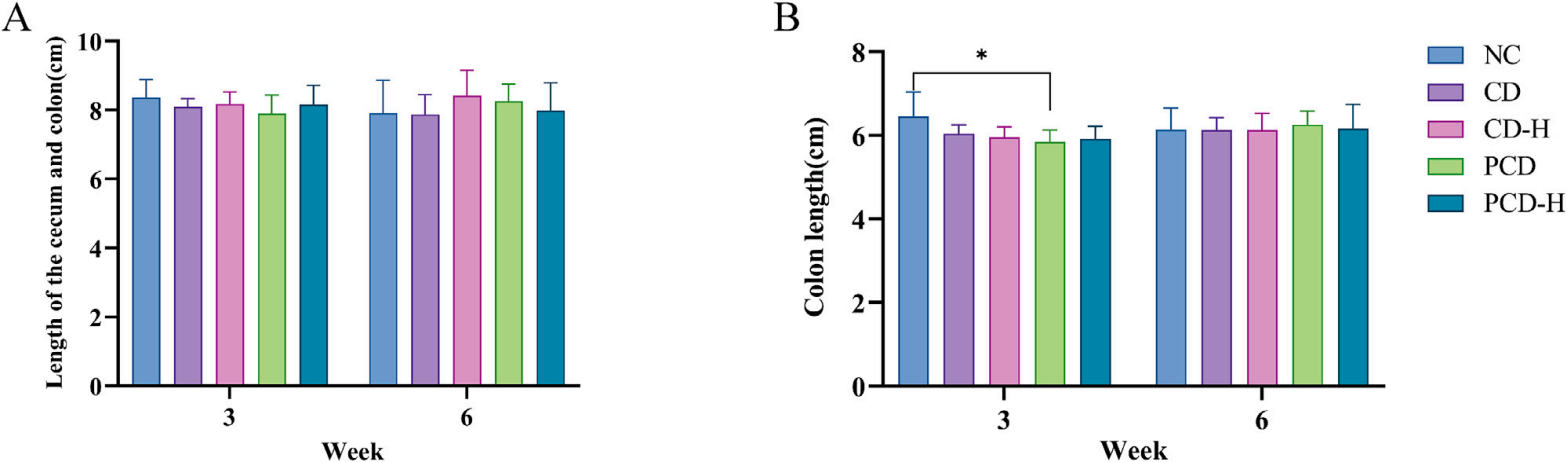
Effects of CD and PCD on cecal and colon lengths in mice **(A)** Length of the cecum and colon. **(B)** colon length. Data were represented as the mean ± standard deviation (n = 6). *p < 0.05.

In this study, the effects of CD and PCD on cecal and colon length in mice were measured (Supplementary Figure S3). As shown in Figure 4A, the total length of the cecum and colon did not change significantly with increasing administration time and dose. In Figure 4B, the length of the colon in the dosed group decreased to varying degrees after 3 weeks of dosing compared to the NC group. However, there was no significant changes occurred after 6 weeks of administration.

### 3.6 Effect of CD and PCD on pathologic changes in the mouse colon

**FIGURE 5:**
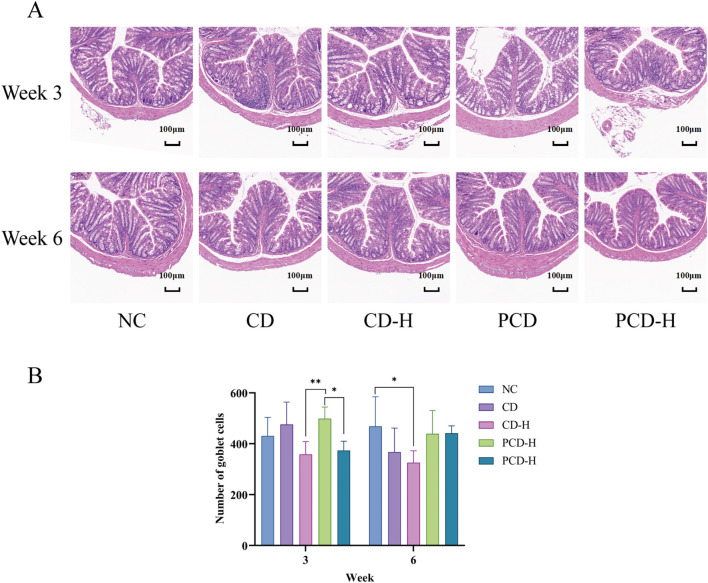
Effects of CD and PCD on pathological changes. **(A)** Colon. (Magnification: ×100) **(B)** Number of goblet cells. Data were represented as the mean ± standard deviation (n = 6). *p < 0.05, **p < 0.01.

In Figure 5A, the colonic epithelial muscular layer and mucosal structure of the NC group were intact, with no edema or inflammatory infiltration in the field of view, and the glands and cupped cells were closely and neatly arranged. After 3 weeks of administration, the goblet cells of the colon in mice were partially ruptured and inflammatory infiltrates were appeared, and the basal crypts were partially damaged. After 6 weeks of administration, the goblet cells of the colon in mice were atrophied and inflammatory infiltrates decreased. As shown in Figure 5B, compared with the PCD group, the number of goblet cells in the colon gland part of the mouse in the CD-H group and PCD-H group was significantly reduced after 3 weeks of administration (*p* < 0.05). Compared with the NC group, the number of goblet cells in the CD-H group was significantly reduced after 6 weeks of administration (*p* < 0.05).

### 3.7 Effects of CD and PCD on the levels of IL-6, IFN-γ, and TNF-α in mice

**FIGURE 6:**
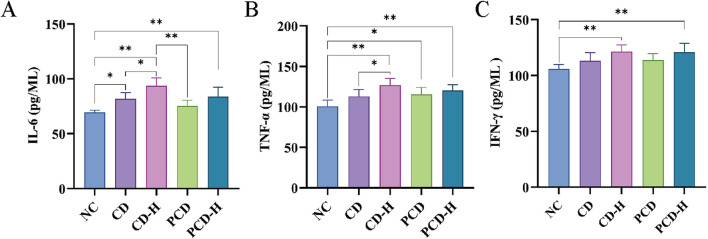
Effects of CD and PCD on Inflammatory factors in healthy mice. **(A)** IL-6. **(B)** TNF-α **(C)** IFN-γ. Data were represented as the mean ± standard deviation (n = 6). *p < 0.05, **p < 0.01.

As shown in Figure 6, compared to the NC group, the levels of inflammatory factors in serum of mice were increased by CD. The levels of IL-6, IFN-γ, and TNF-α were increased by CD and PCD. The high-dose group exhibited a more significant increase compared to the low-dose group. Compared with the CD group, PCD alleviated the upward trend of IL-6, IFN-γ, and TNF-α.

### 3.8 Effects of CD and PCD on IHC and WB of small intestine in mice

**FIGURE 7:**
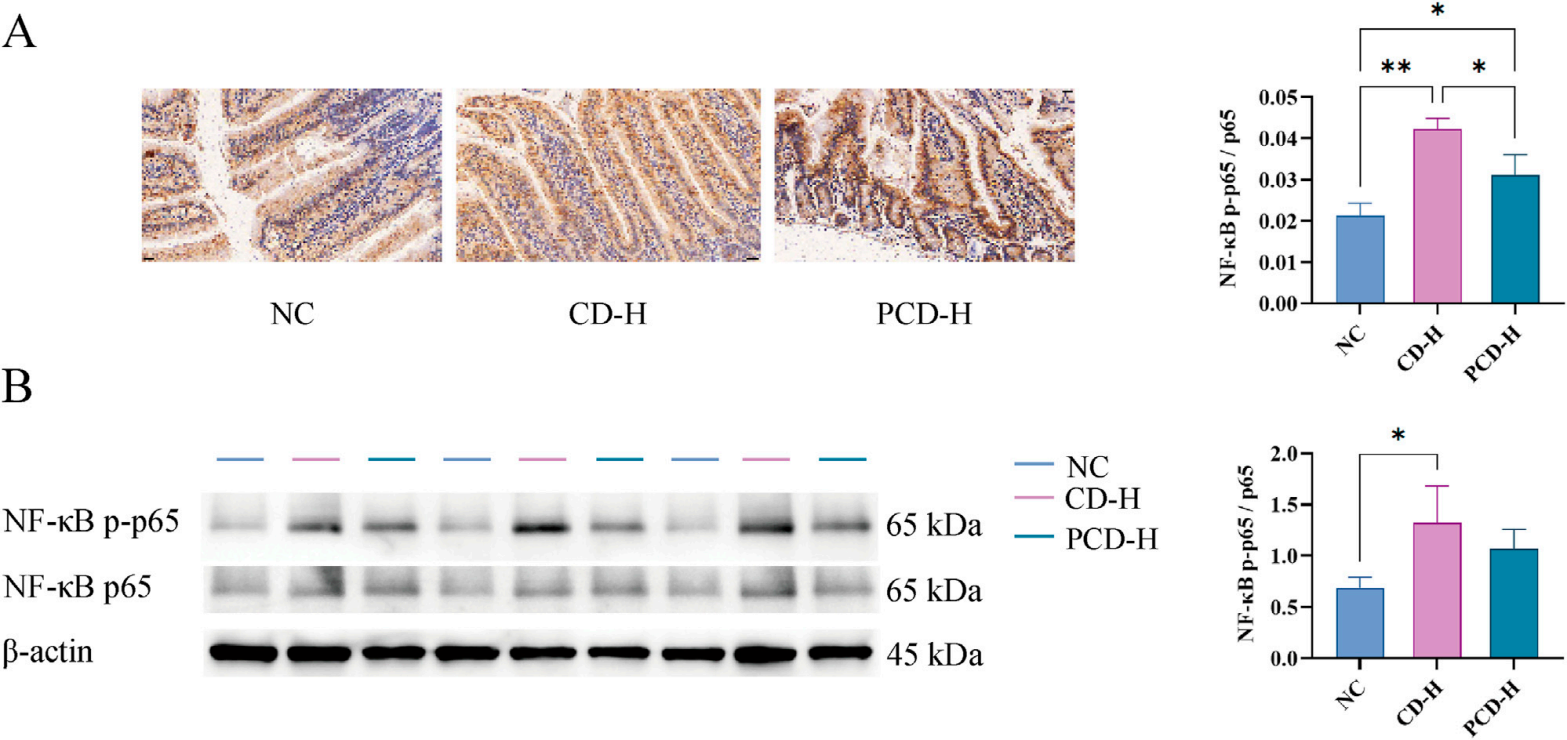
Effects of CD and PCD on IHC and WB of small intestine in mice. **(A)** IHC analysis. (Magnification: ×400) **(B)** Expression of NF-κB p-p65, NF-κB p65 and β-actin of the small intestine in each group tested by WB analysis. Data were represented as the mean ± standard deviation (n = 3). *p < 0.05, **p < 0.01.

As shown in Figure 7, compared with NC group, the expression of NF-κB p65 in CD-H group and PCD-H group was significantly increased, and the expression of NF-κB p65 in CD-H group was more obvious than that in PCD-H group.

### 3.9 Effects of CD and PCD on SCFAs in mice

**FIGURE 8:**
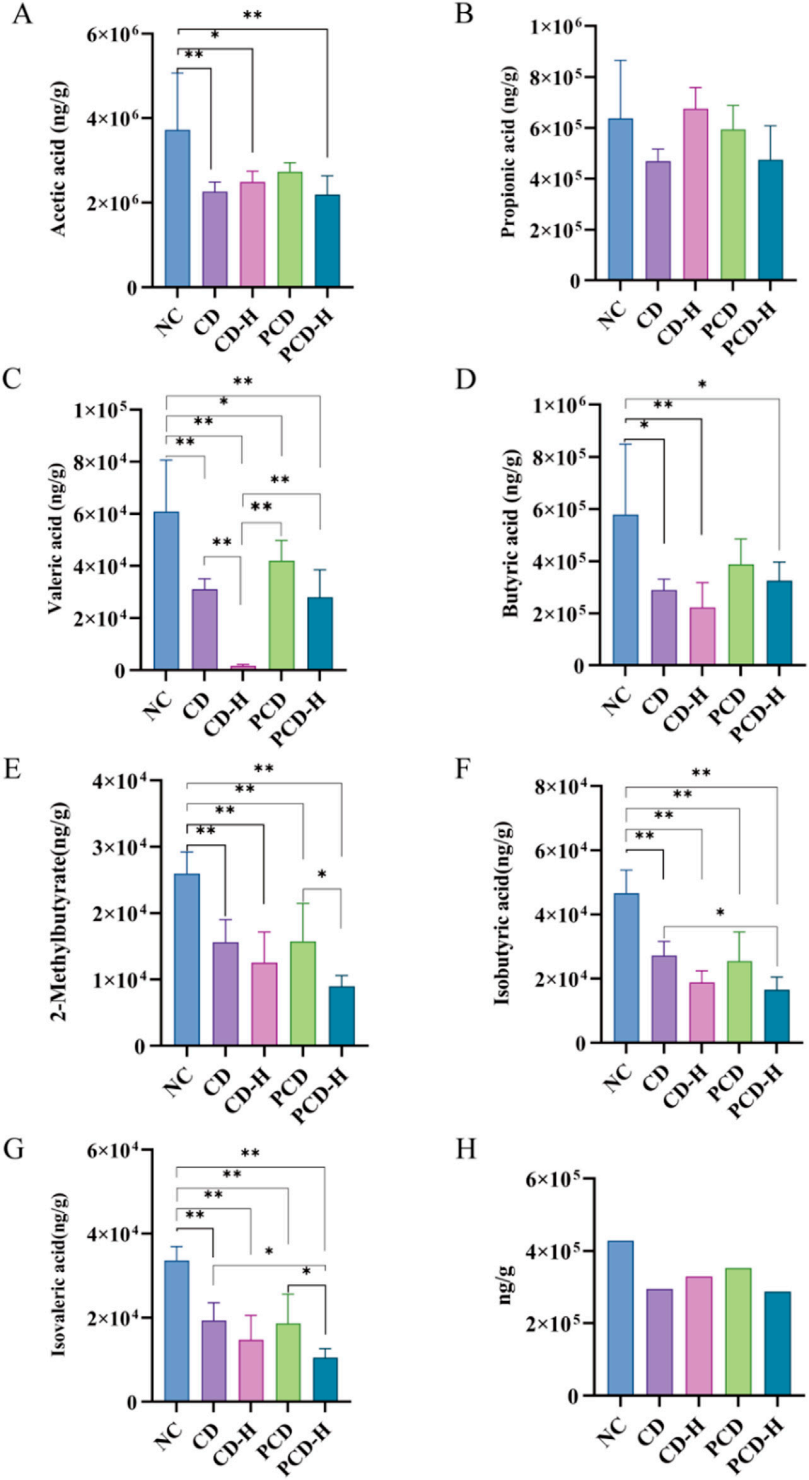
Effects of CD and PCD on SCFAs in healthy mice. **(A)** acetic acid. **(B)** Propionic acid. **(C)** Valeric acid. **(D)** Butyric acid. **(E)** 2-Methylbutyrate. **(F)** Isobutyric acid. **(G)** Isovaleric acid. **(H)** Total SCFAs. Data were represented as the mean ± standard deviation (n = 6). *p < 0.05, **p < 0.01.

The linear regression equations and correlation coefficients were shown in Supplementary Table S1. As shown in Figure 8, compared to the NC group, the content of SCFAs in feces of mice was reduced by CD. The contents of acetic acid, propionic acid, butyric acid, valeric acid, isobutyric acid, 2-methylpenteric acid and isovaleric acid in feces were reduced by CD and PCD. The high-dose group exhibited a more significant decrease compared to the low-dose group. Compared with the CD group, PCD alleviated the downward trend of acetic acid, butyric acid, and valeric acid.

### 3.10 CD and PCD regulated GM in mice

#### 3.10.1 Effects on GM diversity and richness

**FIGURE 9:**
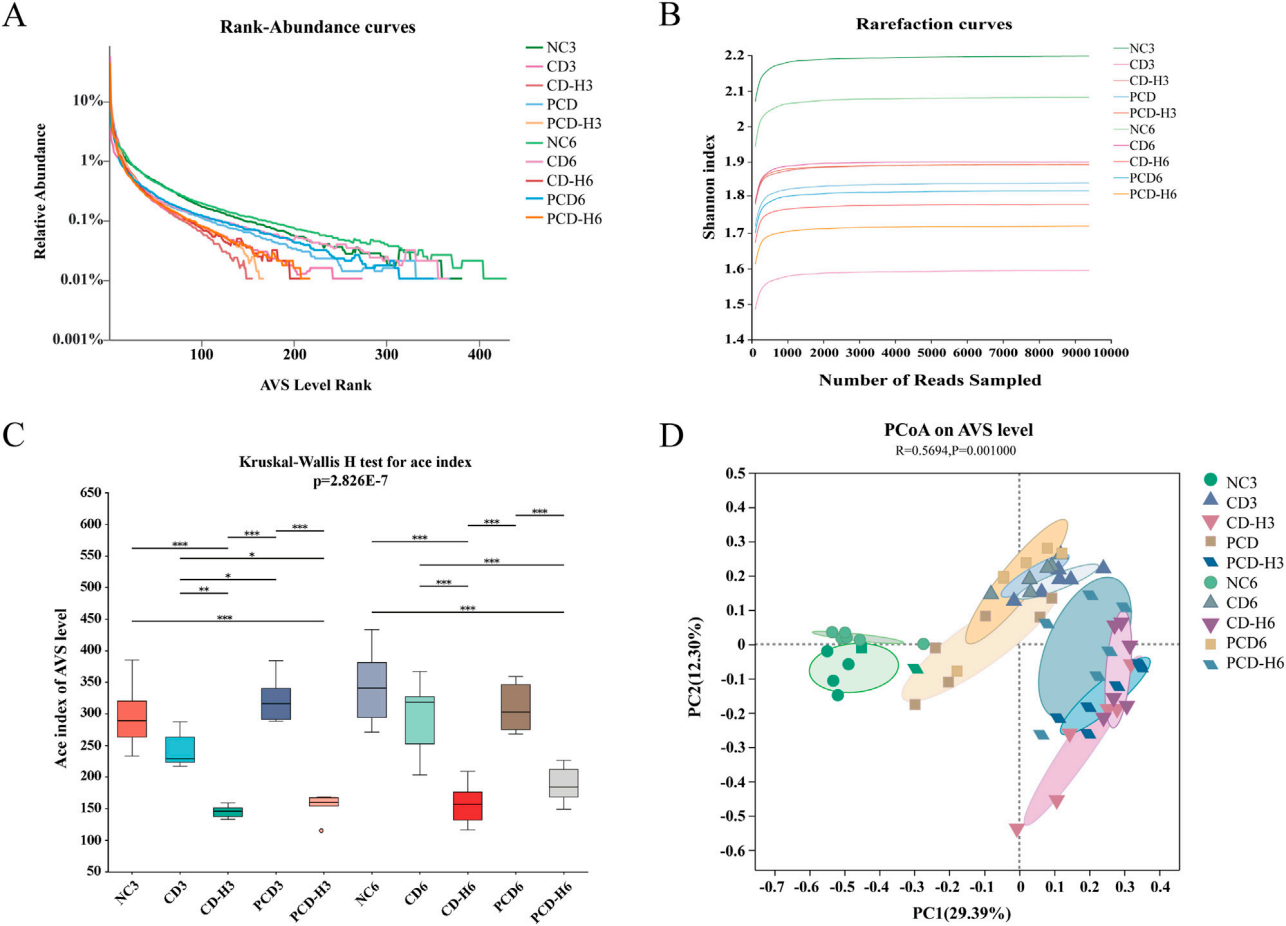
Effect of CF and CP on the diversity of intestinal microorganisms in mice. **(A)** Rank-Abundance curves. **(B)** Rarefaction curves. **(C)** Alpha. **(D)** PCoA. Data were expressed as means ± SD (n = 6). *p < 0.05, **p < 0.01, ***p < 0.001.

Rank-Abundance curves were used to demonstrate species richness and community uniformity. As shown in Figure 9A, in the horizontal direction, the NC group had a wider curve width compared to the administered group, and the PCD group had a wider curve width compared to the CD group, i.e., CD decreases the abundance of the GM, whereas PCD can alleviate the degree of its decrease; the NC group had a more gradual curve compared to the administered group, and the PCD group had a more gradual curve compared to the CD group, which shows that CD decreases the homogeneity of the GM, and PCD alleviates this change. In Figure 9B, the rarefaction curves of all groups tended to be flat, indicating that the amount of data sequenced is sufficient to basically reflect most of the information about the microbial diversity in the samples. In Figure 9C, there was a significant difference in richness and diversity between groups in Alpha diversity analysis, and there was an overall decreasing trend in species diversity of the bacterial flora in the administered group compared to the NC group, which was mitigated by PCD compared to CD. GM species richness and diversity also tended to decrease with increasing administered dose. In Figure 9D, PCoA analysis showed that the sample distances between the same groups were small, which indicated that the structural distribution of the GM was compact and similar. The composition of the intestinal bacteria in both the CD and PCD groups was different from that of the NC group, with the difference in the low-dose group being smaller than that in the high-dose group; and the PCD group was closer to the normal group than the CD group, and its difference was smaller than that of the CD.

#### 3.10.2 Effects of CD and PCD on GM structure and composition

**FIGURE 10:**
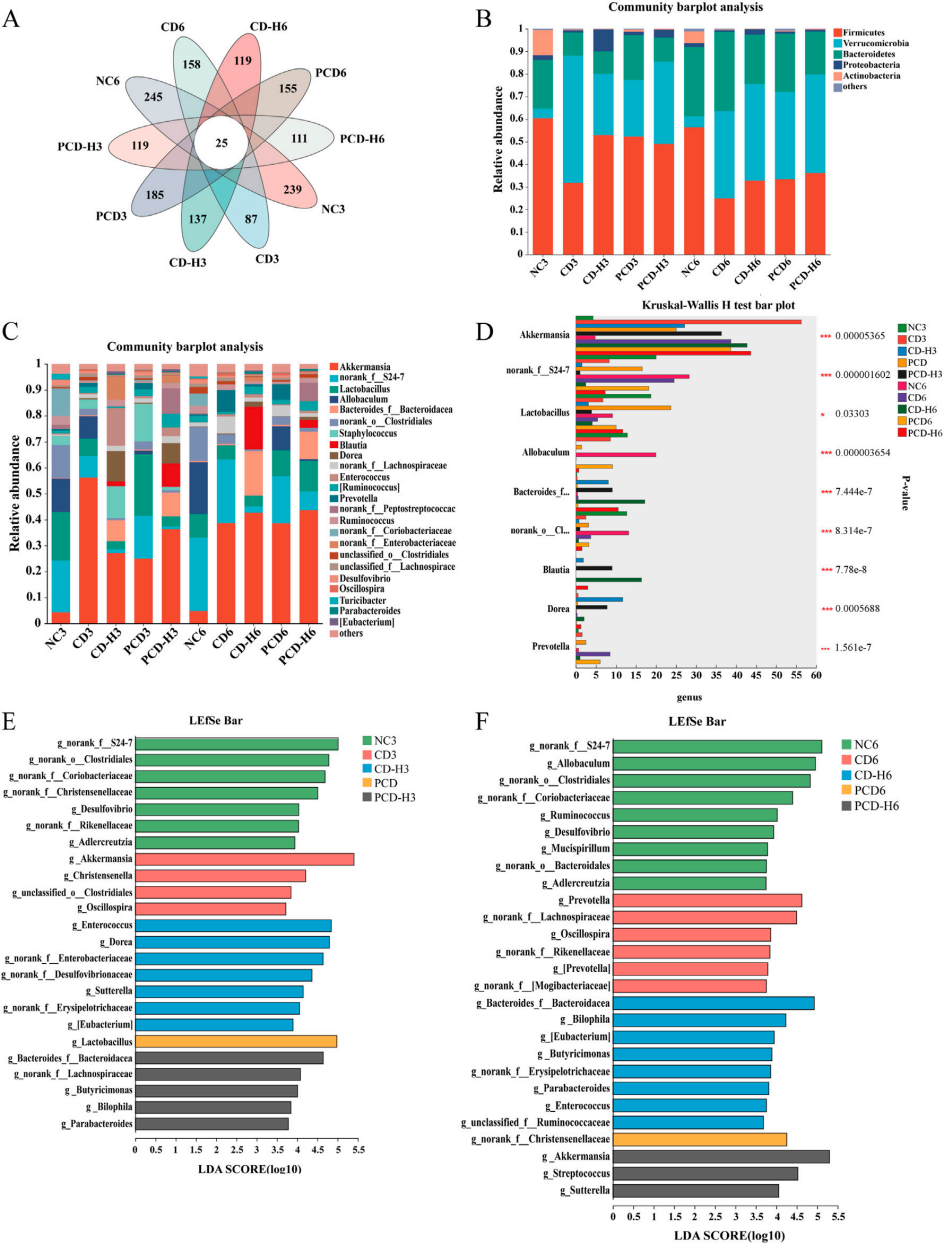
Effect of CD and PCD on the relative abundance of GM in mice. **(A)** venn. **(B)** phylum. **(C)** genus. **(D)** Multi-group species variability analysis. **(E)** Week 3 genus level LEfSe analysis. **(F)** Week 6 genus level LEfSe analysis. Data were expressed as means ± SD (n = 6). *p < 0.05, **p < 0.01, ***p < 0.001.

As shown in Figure 10A, the species Venn diagram, reflecting the similarity and overlap of species composition between groups, showed that the bacterial colony structure varied greatly between groups. Compared with the NC group, the total number of AVS of the bacterial colonies decreased after administration of the drug, with the decrease in the CD group being more pronounced than that in the PCD group.

As shown in Figure 10B, the dominant species in each group at the phylum level were Firmicutes, Verrucomicrobia, and Bacteroidetes, with a relative abundance of more than 85%, followed by Proteobacteria and Actinobacteria, among others. The experimental study found that CD reduced the proportion of Firmicutes, and the decreasing trend increased with the prolongation of the administration time, in which the decreasing trend was obvious in the CD group compared with the PCD group; the proportion of Verrucomicrobia in the administration group increased, and the degree of elevation in the CD group was greater than that in the PCD group; the proportion of Bacteroidetes in the administration group firstly decreased, and then gradually increased.

As shown in Figure 10C, at the genus level the groups were mainly present in the genera *Akkermansia*, *Lactobacillus*, *Allobaculum* and *Bacteroides*. Compared with the NC group, the proportion of *Akkermansia* was elevated by CD and the upward trend was more significant in the CD group than in the PCD group; *Lactobacillus* was reduced by CD and PCD mitigated this reduction; and the proportion of *Allobaculum* was decreased in the administered group, and the reduction in the CD group was progressively greater than that in the PCD group with the extension of time.

The analysis of species differentiation at the genus level in multiple groups was shown in Figure 10D, which showed that the abundance of *Akkermansia* was increased in the administered group compared to the NC group; and the abundance of *norank_f_S24-7* was decreased, with a more pronounced effect in the CD group than in the PCD group; the abundance of *Lactobacillus* and *Allobaculum* was reduced; and the abundance of *Bacteroides* was increased; the abundance of *norank_o_Clostridiales* was decreased, in which the decreasing trend of CD was more obvious than that of PCD; and the abundance of *Blautia*, *Dorea*, and *Prevotella* was increased.

LEfSe analyses, shown in Figures 10E, F, were used to mine for species that differed significantly between groups, and linear discriminant analysis (LDA) scores >3 were selected as the screening criterion used to identify microorganisms with higher relative abundance in each group.

In the third week, the genus level dominant strains in the NC group included *norank_f_S24-7*, *norank_o_Clostridiales*, *norank_o_Coriobacteriaceae*, *Desulfovibrio*, etc.; genus level dominant strains in the CD group included *Akkermansia*, *unclassified_o_Clostridiales*, *Christensenella* and *Oscillospira*. CD-H group genus level dominant species include *norank_f_Enterobacteriaceae*, *Enterococcus*, *Dorea* etc.; PCD group genus level dominant species include *Lactobacillus*; PCD-H group genus level dominant species include Butyricimonas, *Bilophila*, *Parabacteroides*, *norank_f_Lachnospiraceae* and *Bacteroides_f_Bacteroidacea*. In the sixth week, the genus level dominant strains in the NC group included *Allobaculum*, *norank_f_Coriobacteriaceae*, *norank_f_S24-7*, *norank_o_Clostridiales*, etc.; in the CD group the genus level dominant strains included *Prevotella*, *norank_f_Lachnospiraceae*, *Oscillospira*, etc.; CD-H group genus level dominant species include *Bilophila*, *Bacteroides_f_Bacteroidacea*, *Butyricimonas*, etc.; PCD group genus level dominant species include *norank_f_Christensenellaceae*; PCD-H group genus level dominant species include *Akkermansia*, *Sutterella* and *Streptococcus*.

In summary, the result of CD affecting the GM composition of healthy mice was demonstrated by community composition analysis. At the phylum level, CD decreased the proportion of Firmicutes and increased the proportion of Verrucomicrobia, at the genus level elevated the proportion of *Akkermansia* in the administered group and decreased the proportions of *Lactobacillus* and *Allobaculum*, however, PCD mitigated this decreasing trend in comparison to CD. During the administration period, the dominant genera were different in the NC and dosing groups, and the composition of the dominant genera species was altered by CD and PCD, and the dominant genera of each group changed over time.

### 3.11 Correlation analysis between SCFAs and GM

**FIGURE 11:**
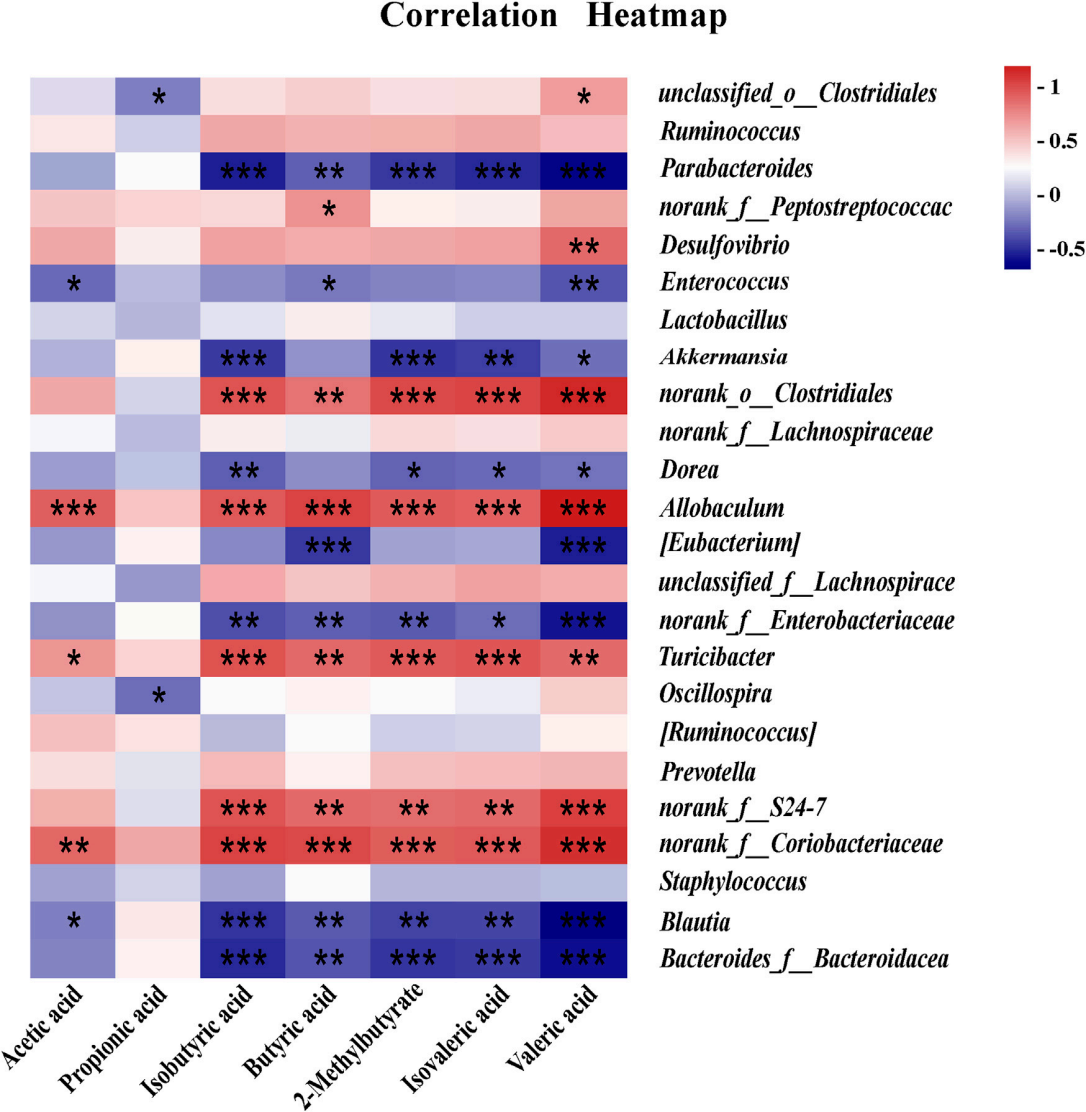
Correlation analysis between SCFAs and GM. *p < 0.05, **p < 0.01, ***p < 0.001.

The correlation analysis of GM and SCFAs suggested that there was a significant positive correlation between GM (*Allobaculum and norank_f_S24-7*) and SCAFs (Isobutyric acid, 2-Methylbutyrate, Butyric acid, Isovaleric acid and Valeric acid) at the genus level, and there was a significant negative correlation between GM (*Akkermansia and Bacteroides*) and SCAFs (Isobutyric acid, 2-Methylbutyrate, Isovaleric acid and Valeric acid) at the genus level. In addition, there was a negative correlation between *Lactobacillus* and SCFAs without a significant difference (Figure 11).

## 4 Discussion

In this study, CD was found to affect the composition of the GM in healthy mice, reduced the diversity of GM and the content of SCFAs. In addition, the bidirectional modulating effect of CD was confirmed, which had both laxative and astringent effects, and PCD produced a milder effect relative to CD.

Symptoms of diarrhea were that the feces pass through the gastrointestinal tract for a shorter period of time, the stools become soft or the feces are watery; the primary symptom of constipation is prolonged defecation time, less water in feces, dry and hard stools (Marques et al., 2023; Wen et al., 2023). Diarrhea and constipation were accompanied by damage to the gastrointestinal tract such as destruction of gastrointestinal villi and inflammatory infiltrates, and the causes of gastrointestinal damage are related to the release of inflammatory factors such as IL-6 and TNF-α (Seyedmirzaee et al., 2016; Yao et al., 2021). Meanwhile, studies have shown that the occurrence of intestinal inflammation is related to the regulation of PI3K/Akt/NF-κB signaling pathway (Yan et al., 2022). Biological factors such as cytokines or viruses can lead to phosphorylation of PI3K to activate Akt, and the activated Akt can then activate NF-κB to regulate the expression of IFN-*γ*, TNF-α or other inflammatory factors (Rahmani et al., 2020; Choi et al., 2022). Our study showed that the administration of CD initially induced diarrhea in mice, which later progressed to constipation with prolonged administration, with significant changes in fecal water content and intestinal transit time. The HE stained sections of the gastrointestinal tract show that CD causes damage to the gastrointestinal tract, and the study found that the expression of NF-κB and inflammatory factors were increased after producing mild constipation in the sixth week. However, PCD produced weaker effects than CD.

SCFAs were the end products of fermentation of dietary fiber by GM, mainly including acetic acid, propionic acid and butyric acid (Tan et al., 2023). They served several essential functions, including the maintenance of intestinal mucosal integrity, enhancement of immune system responses, promotion of intestinal electrolyte balance, regulation of gut microbiota, and resistance to inflammation (Rauf et al., 2022). Impaired intestinal epithelial integrity can lead to chronic low-grade inflammation. However, acetic and butyric acids were considered to enhance epithelial integrity (Furusawa et al., 2013; Tong et al., 2016), and acetic acid could promote intestinal motility (Niwa et al., 2002). It has been found that the levels of butyric acid reduced lead to increased oxygenation thereby promoting microecological dysregulation, and enhancing the expansion of aerobic pathogens in the gut (Handa et al., 2023). In the present study, it was found that CD reduced the contents of acetic acid, propionic acid, butyric acid, valeric acid, isobutyric acid, 2-methylpentanoic acid and isovaleric acid in feces of mice.

The growing evidence suggested that the balance of GM played an important role in the development of human health (Adak and Khan, 2019; Kataoka, 2016; Yang Y. et al., 2023). Both Coptidis Rhizoma and its formulation had been shown to regulate the composition of GM (Chen et al., 2018; Yang F. et al., 2023). The results of this study showed that the abundance of Firmicutes decreased, the proportion of Verrucomicrobia increased, and Bacteroidetes first decreased and then gradually increased in the administered group compared to the normal group. These changes may further lead to some bacterial imbalances. For example, we found that at the genus level *Akkermansia*, *Dorea*, *Bacteroides*, and *Blautia* were upregulated in the CD group compared to the NC group, while the levels of *Lactobacillus*, *Allobaculum*, *Ruminococcus*, and *norank_f_S24-7* were reduced. It is noteworthy that PCD can alleviate the GM dysbiosis induced by CD. The increased abundance of *Akkermansia* can exacerbate the occurrence and development of intestinal inflammatory reactions (Castro-Mejía et al., 2016). Excess *Bacteroides* increases mucus degradation, thereby reducing intestinal inflammation, especially the colonic mucus barrier, by reducing bacterial interactions with intestinal epithelial cells (Desai et al., 2016). *Lactobacillus* is the main probiotic in the gut and plays a crucial role in maintaining the microecological balance of the gastrointestinal tract (Huang et al., 2022). The relative abundance of *Allobaculum* had been reported to be positively correlated with the levels of RORγt in the ileum (Cox et al., 2014).

CD regulates short-chain fatty acid metabolism by affecting the composition of GM in healthy mice, reducing the abundance and homogeneity of GM. These had been partially validated in correlation analyses between gut flora and SCFAs. For example, there was a significant positive correlation between *Allobaculum* and SCFAs (isobutyric, 2-methylbutyric, butyric, isovaleric, isovaleric, and valeric acids) and a significant negative correlation between GM (*Akkermansia* and *Bacteroides*) and SCFAs (isobutyric, 2-methylbutyric, isovaleric, and valeric acids). Additionally, there was a negative correlation between *Lactobacillus* and SCAFs with no significant difference.

Numerous studies have shown that GM affects intestinal immune homeostasis, with SCFAs being the primary mediators (Zhang S. et al., 2023). GM was found to activate T cells and induce the production of inflammatory factors through its metabolite SCFAs. In addition, SCFAs inhibited IL-6 and TNF-α production by monocytes, myeloid dendritic cells, and plasmacytoid dendritic cells (Porbahaie et al., 2023). Butyrate reduces the development of interferon-gamma (IFN-γ) generating cells while promoting the development of regulatory T cells (Duan et al., 2023). Decrease in *Lactobacillus* can lead to the production of TNF-α and IFN-γ and induce IL-6 and IL-23 infiltration into helper T cells 17 (Th17), thus inducing an inflammatory response (Gerassy-Vainberg et al., 2018). It was shown that CD and PCD decreased the levels of SCFAs and elevated levels of TNF-α, IL-6 and IFN-γ, and the effect of CD was more obvious. In summary, PCD alleviated the gastrointestinal injury and abnormal levels of inflammatory factors caused by CD, which are related to PCD’s regulation of GM disorders and GM mediated SCFA changes.

PCD is a processed product of CD. According to the theory of TCM, CD may cause gastrointestinal damage due to side effects if used for a long period of time or in large quantities, so it is necessary to process it so as to mitigate the effects and alleviate the side effects. This study also showed that PCD was weaker than CD in causing damage to the gastrointestinal tract of healthy mice. For example, PCD ameliorates CD-induced weight loss as well as histopathological damage to the gastrointestinal tract and levels of inflammatory factors. Moreover, CD-H showed a tendency to exacerbate the inflammatory damage in the gastrointestinal tract induced by CD. These findings suggest that PCD can reduce the side effects of CD and increase the range of safe therapeutic doses for CD. This is consistent with the theory that processing of Chinese herbal medicines can reduce their toxicity and maintain their efficacy. These findings suggested that processing of CD can reduce its side effects.

In general, the results of the present study indicated that CD can affect the balance of GM in healthy mice by modulating the abundance of *Akkermansia*, *Allobaculum* and *Bacteroides*. However, only correlation analyses of SCFAs and GM were performed in this study, and further causal link has not been established. Therefore, follow-up studies will screen differential strains based on macro-genome sequencing technology, and then compare and analyze their effects on mouse GM under the intervention of the differential strains, so as to elucidate the causal relationship between the side effects induced by CD and the alteration of intestinal flora.

## 5 Conclusion

The current study demonstrated that CD can cause mild diarrhea and constipation, which may be associated with GM disorders and GM-mediated changes in SCFAs, as well as the anomalous expression of NF-κB and the release of inflammatory factors, and induce pathological intestinal damage, and the effects of PCD were weaker than that of CD. Furthermore, the alleviating effect of PCD on gastrointestinal injury may be due to the differences in the types and contents of ingredients in PCD. And this study contributes to our understanding of the different effects of CD and PCD on the intestine, and provides a reference for safe and rational use of CD in clinics.

## Data Availability

The datasets presented in this study can be found in online repositories. The names of the repository/repositories and accession number(s) can be found in the article/Supplementary Material.
