# Supplementary material for: Effect of Coptidis Rhizoma on gastrointestinal system before and after processing with wine based on gut microbiota and short chain fatty acids
Source: Front Pharmacol. 2024 Nov 18;15:1492047. doi: 10.3389/fphar.2024.1492047 (PMC11608971; doi:10.3389/fphar.2024.1492047)
Supplement: Supplementary file 1 [file DataSheet1.docx]

Supplementary data

Fig. S1. Total ion flow diagram for UPLC-ESI-MS/MS analysis of CD and PCD.

(A) Negative ion mode of CD, (B) Positive ion mode of CD, (C) Negative ion mode of PCD, (D) Positive ion mode of PCD.


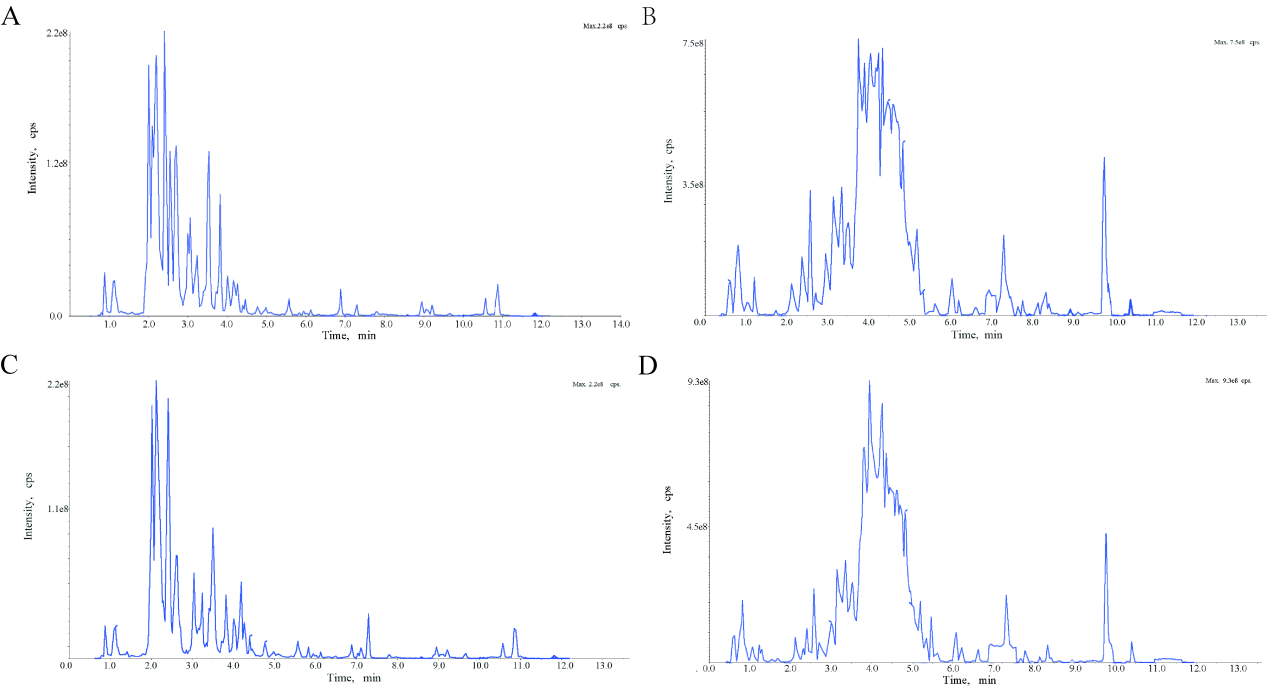


Fig. S2. Raw data of WB.

(A) NF-κB p-p65, (B) NF-κB p65, (C) β-actin.


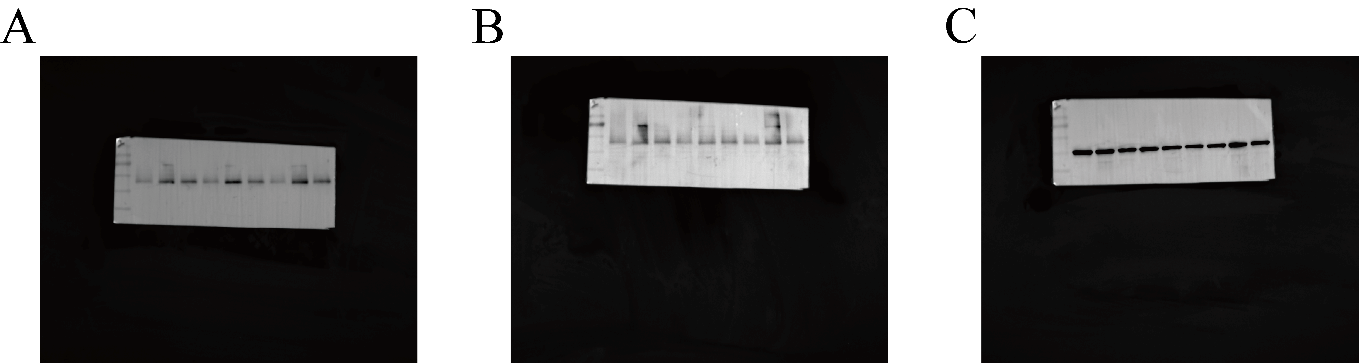
Fig. S3. Pictures of colon and cecum.


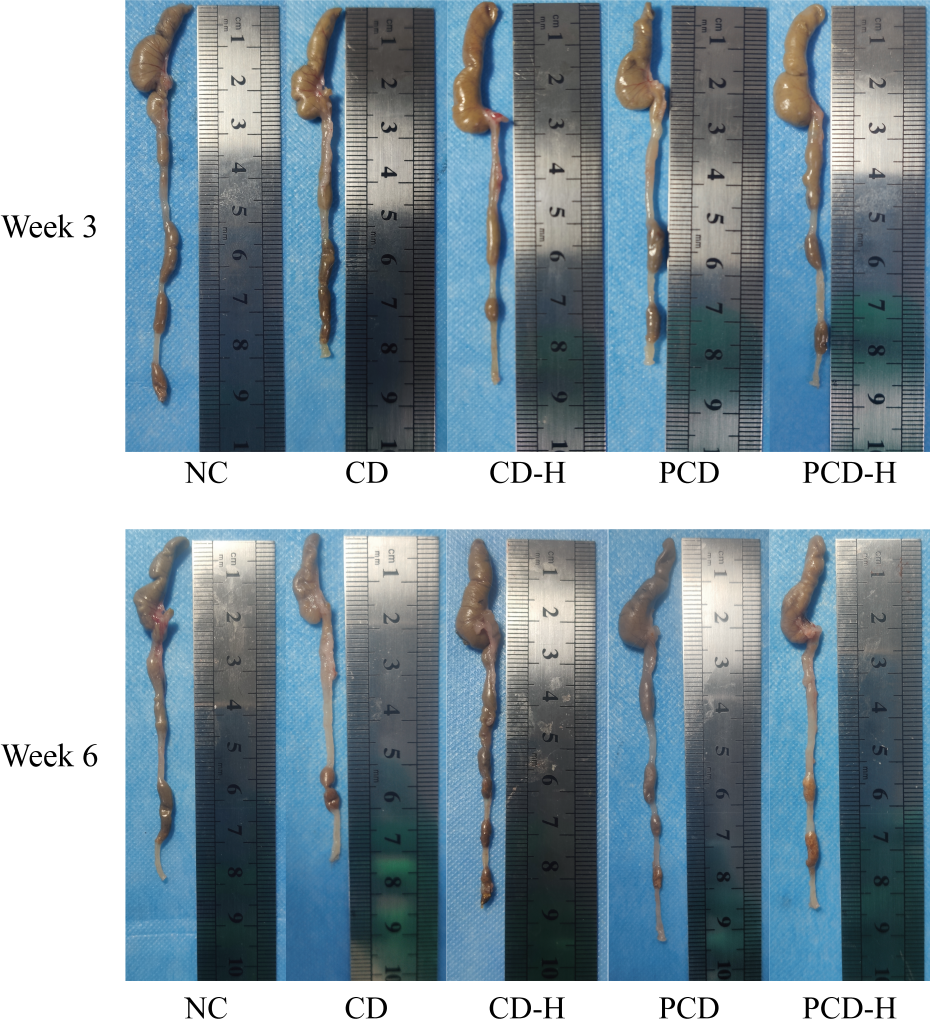


Table S1. Standard curves of different SCFAs and comparison of SCFAs content in each group. (n=6)

Table S1. Standard curves of different SCFAs and comparison of SCFAs content in each group

| Compounds name | Regression equation | Correlation coefficient | NC  (ng/g) | CD  (ng/g) | CD-H  (ng/g) | PCD  (ng/g) | PCD-H  (ng/g) |
| --- | --- | --- | --- | --- | --- | --- | --- |
| Acetic acid | Y=7.408e-3X+4.116e-3 | 0.995 | 3723955±1345307 | 2260583±225111 | 2490490±252266 | 2734954±206967 | 2190871±440351 |
| Propionic acid | Y=9.418e-3X-8.223e-3 | 0.996 | 637441±228074 | 468927±47190 | 675409±82399 | 593965±94332 | 474798±132702 |
| Isobutyric acid | Y=9.588e-3X-9.974e-3 | 0.997 | 46648±7137 | 27210±4404 | 20885±4970 | 25460±9040 | 16530±3906 |
| Butyric acid | Y=7.455e-3X-1.147e-2 | 0.996 | 578579±270579 | 289532±41473 | 222503±95805 | 387170±97067 | 325256±71312 |
| 2-Methylbutyrate | Y=1.113e-2X-5.425e-3 | 0.997 | 25947±3255 | 15609±3412 | 12546±4608 | 15753±5695 | 8990±1619 |
| Isovaleric acid | Y=1.067e-2X-1.385e-2 | 0.996 | 33632±3277 | 19332±4220 | 14764±5797 | 18648±6924 | 10502±2134 |
| Valeric acid | Y=1.094e-2x-1.169e-2 | 0.996 | 60883±19783 | 31092±3991 | 1682±566 | 41976±7850 | 28081±10448 |
